# Supplementary material for: Precise Design of Alginate Hydrogels Crosslinked with Microgels for Diabetic Wound Healing
Source: Biomolecules. 2022 Oct 28;12(11):1582. doi: 10.3390/biom12111582 (PMC9687833; doi:10.3390/biom12111582)
Supplement: Supplementary file 1 [file biomolecules-12-01582-s001.zip › biomolecules-1934603-supplementary.pdf]

Electronic Supporting Information for

Precisely design of alginate hydrogels crosslinked with microgels for diabetic wound healing

Yishu Yan [1,†,*], Panpan Ren [1,†], Qingqing Wu [1], Tianmeng Zhang [2]

1. School of Life Science And Health Engineering, Jiangnan University, Wuxi 214122, China
2. Bloomage Biotechnology Corporation Limited, Jinan 250101, China

* Correspondence to Yishu Yan (E-mail: yanyishu@sina.com). The addresses: No. 1800, Lihu Avenue, Wuxi 214122, China

† The authors contributed equally to this paper.

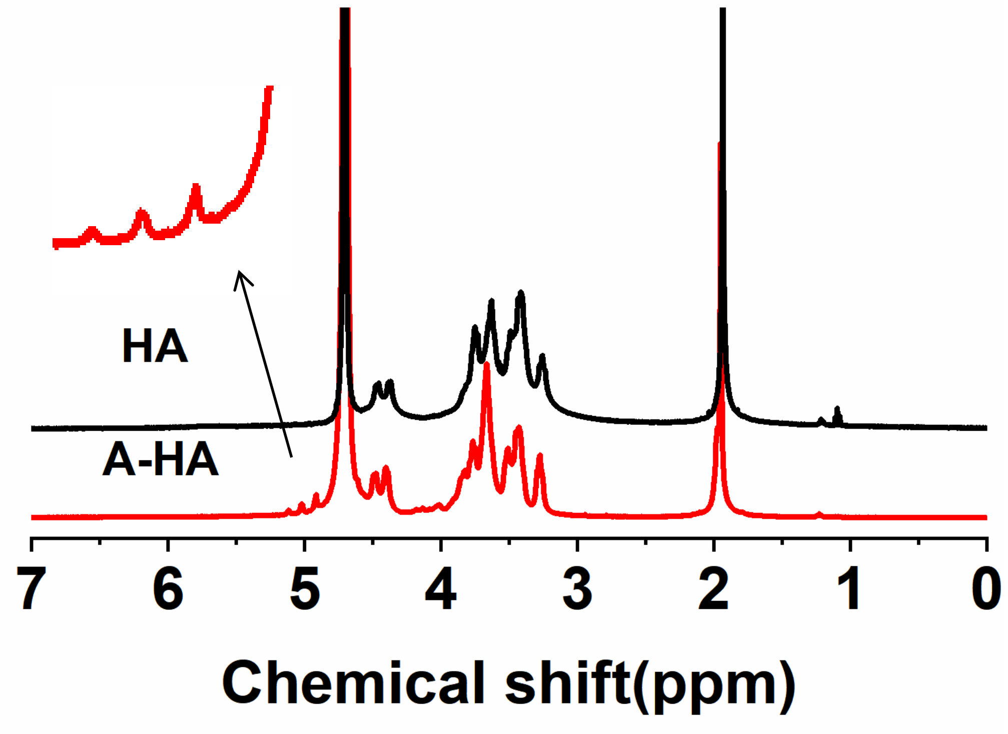

Figure S1 ^{1}H-NMR spectra of HA and A-HA. Compared with HA, two new peaks at 4.9 ppm and 5.0 ppm were observed on the ^{1}H-NMR spectrum of A-HA, corresponding to hemiacetalic protons formed from the aldehyde groups and neighboring hydroxyl groups (Li et al., 2014).

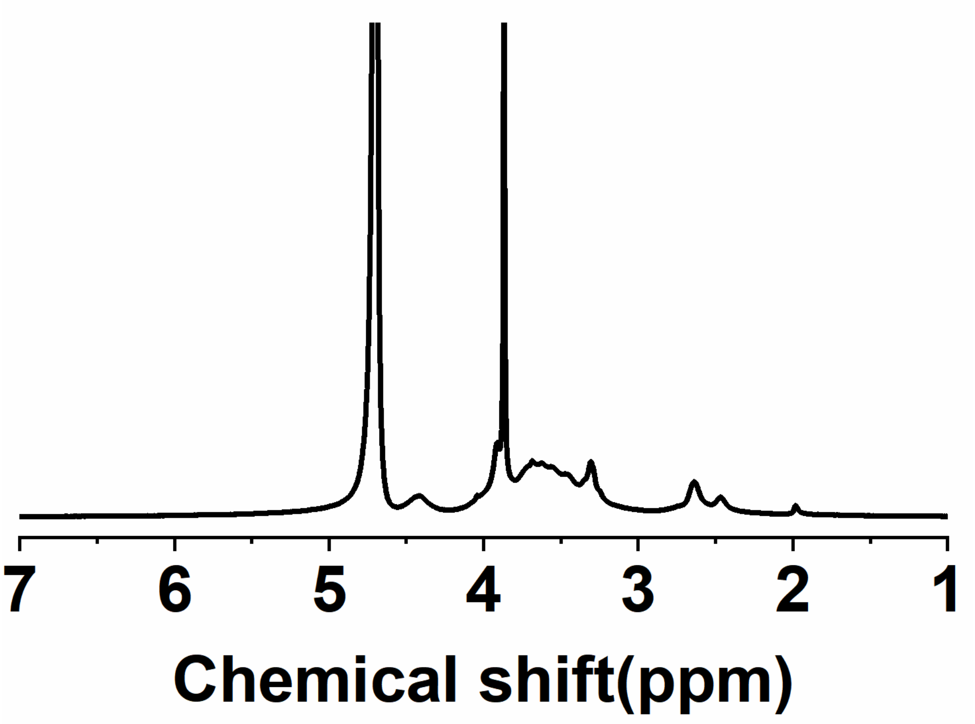

Figure S2 The ^{1}H-NMR spectrum of CMCS. The signals at 2.7 ppm was corresponded to the carboxymethyl group grafted onto the amino groups (C-2) and hydroxyl groups (C-6).

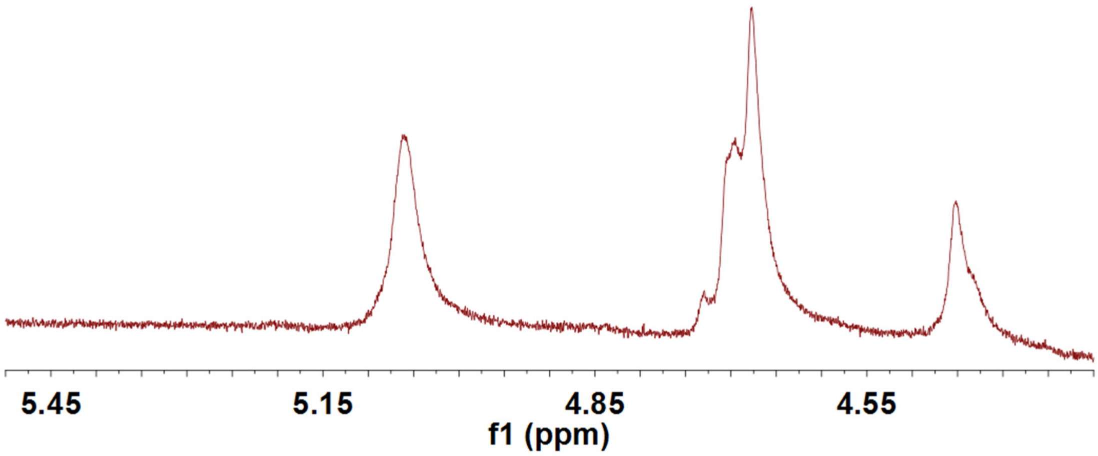

Figure S3 The ^{1}H-NMR spectrum of alginate. The chemical shifts of the anomeric proton signals were A (G) at around 5.2 ppm; B1 (MGM) at 4.75 ppm; B2 (MG) at 4.71 ppm; B3 (MM) at 4.68 ppm and C (GG) at 4.48 ppm. The ratio of G/M is around 1:2 according to the spectrum.

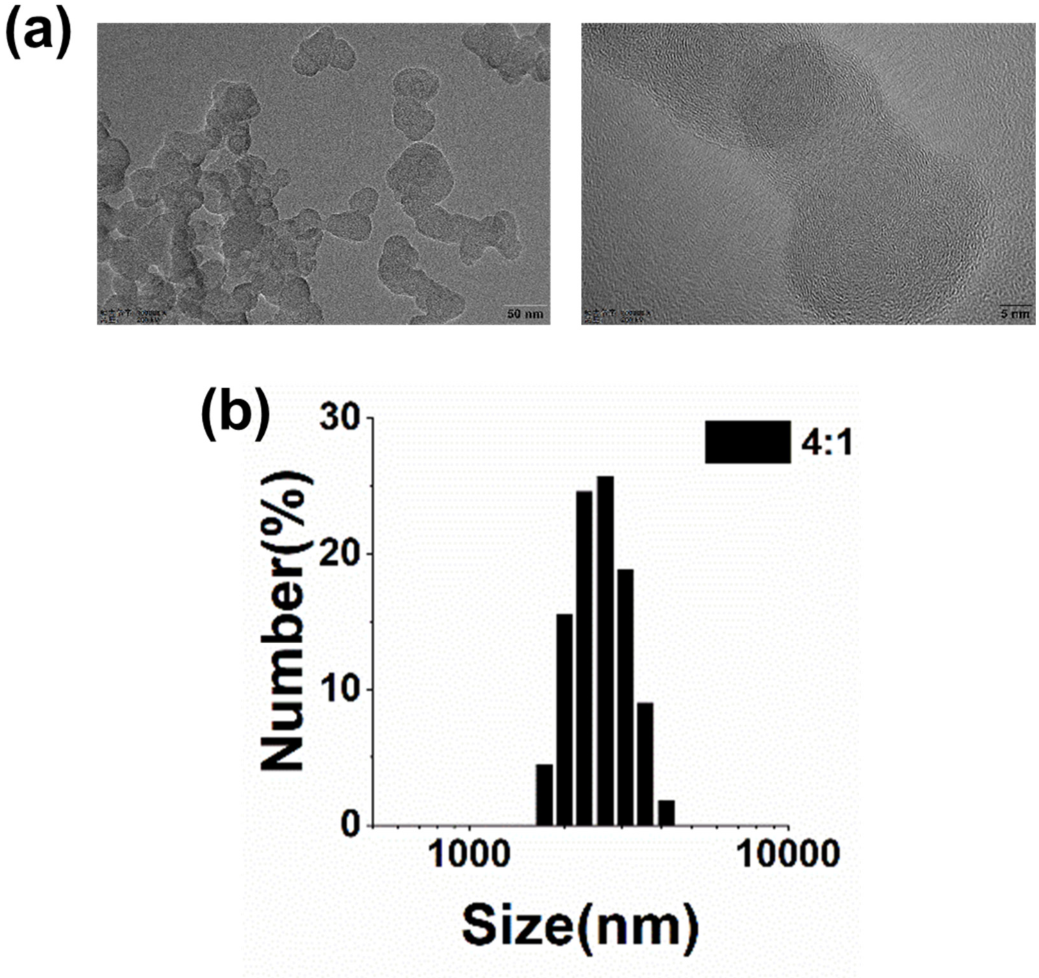

Figure S4 Characterization of the microgels. (**a**) TEM observations of the microgel morphology. (**b**) The dynamic diameter of the microgels.

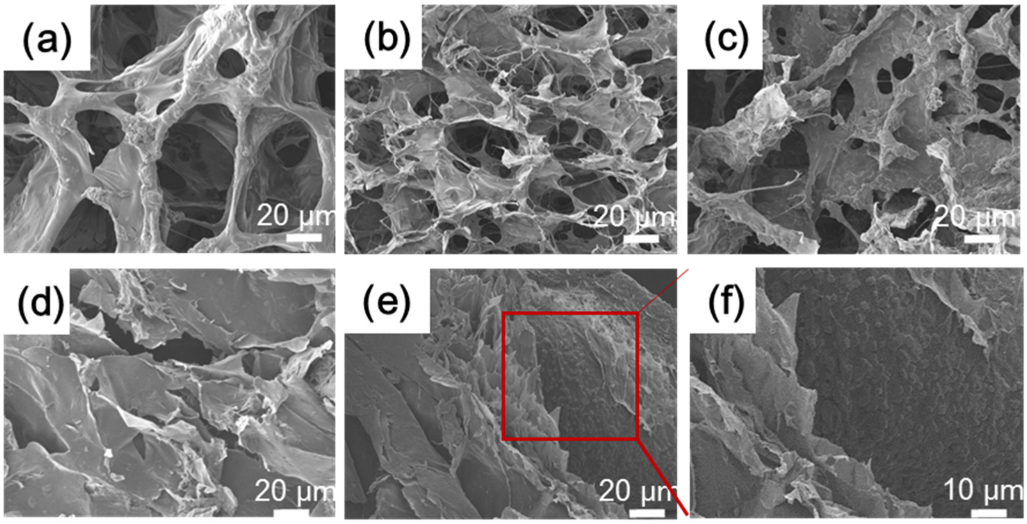

Figure S5 (**a–f**) Enlarged SEM images of the hydrogels of A (**a**), B (**b**), C (**c**), and D (**d**). The images of (**e**) is a side cut of D, local enlarged in (**f**).

Table S1 The relative molecular weight & distribution of the polysaccharides

	**Retention time (Min)**	**Mn （kDa）**	**Mw （kDa）**	**MP （kDa）**
HA	13.33	478500	2873700	1511700
A-HA	14.85	105100	287400	225600
CMCS	16.28	23200	41300	37100

Reference

Li, L., Wang, N., Jin, X., Deng, R., Nie, S., Sun, L., . . . Gong, C. (2014). Biodegradable and injectable in situ cross-linking chitosan-hyaluronic acid based hydrogels for postoperative adhesion prevention. *Biomaterials,* 35(12), 3903-3917.
